# Flavonoids in Plant Salt Stress Responses: Biosynthesis, Regulation, Functions, and Signaling Networks

**DOI:** 10.3390/plants15010171

**Published:** 2026-01-05

**Authors:** Muhammad Tanveer Akhtar, Maryam Noor, Xinyi Lin, Zhaogeng Lu, Biao Jin

**Affiliations:** 1 School of Horticulture and Landscape, Yangzhou University, Yangzhou 225009, China; 2 College of Animal Science and Technology, Yangzhou University, Yangzhou 225009, China

**Keywords:** salinity stress, phenylpropanoid pathway, ROS homeostasis, Na^+^/K^+^ balance, stress signaling, redox buffering, halophytes, metabolic engineering

## Abstract

Soil salinity is a major constraint on global crop production, disrupting photosynthesis, ion homeostasis, and growth. Beyond the roles of classic osmoprotectants and antioxidant enzymes, flavonoids have emerged as versatile mediators of salt stress tolerance at the interface of redox control, hormone signaling, and developmental plasticity. This review summarizes current evidence on how salinity remodels flavonoid biosynthesis, regulation, and function from cellular to whole-plant scales. We first outline the phenylpropanoid–flavonoid pathway, with emphasis on transcriptional control by MYB, bHLH, and NAC factors and their integration with ABA, JA, and auxin signaling. We then discussed how post-synthetic modifications such as glycosylation and methylation adjust flavonoid stability, compartmentation, and activity under salt stress. Functional sections highlight roles of flavonoids in ROS scavenging, Na^+^/K^+^ homeostasis, membrane integrity, and the modulation of ABA/MAPK/Ca^2+^ cascades and noncoding RNA networks. Spatial aspects, including root–shoot communication and rhizosphere microbiota recruitment, are also considered. Based on this synthesis, we propose a flavonoid-centered stress network (FCSN), in which specific flavonoids function as key nodes that connect metabolic flux with hormonal crosstalk and stress signaling pathways. We argue that reconceptualizing flavonoids as central stress network regulators, rather than generic antioxidants, provides a basis for metabolic engineering, bio-stimulant design, and breeding strategies aimed at improving crop performance on saline soils.

## 1. Introduction

As sessile organisms, plants are compelled to confront adverse environmental conditions directly. Climate change has intensified multiple abiotic stresses including drought, flooding, extreme temperatures, and soil salinity that disrupt growth and development and ultimately reduce crop yield and quality [1]. Elevated temperatures increase evapotranspiration, while reduced and erratic rainfall limits leaching, resulting in the progressive accumulation of salts in agricultural soils [2]. Thus, climate change not only generates new saline soils, but also exacerbates existing salinization.

Salinity is now recognized as one of the major abiotic constraints to global food security, impairing plant growth, development, and product quality and causing substantial yield losses [3]. Soil salinization has challenged agriculture for millennia. Projections suggest that, by 2050, up to 50% of arable land may experience salinity problems, particularly in arid and semi-arid regions [4]. At the plant level, salinity initially triggers osmotic stress, followed by toxic ion accumulation (Na^+^, Cl^−^, SO_4_^2−^) [5], leading to poor germination, chlorosis, reduced growth, impaired photosynthesis, nutrient imbalance, electrolyte leakage, and oxidative stress [6,7]. In response, plants initiate coordinated physiological and molecular responses to re-establish ion and osmotic homeostasis while restricting ROS accumulation [8].

Flavonoids are major secondary metabolites that play a critical role in stress mitigation through their anti-oxidative properties, which protect plants from growth inhibition and cell death by reducing oxidative stress through scavenging excess ROS [9]. Flavonoids are the largest group of plant polyphenols and include flavonols, flavones, isoflavones, anthocyanidins, flavanones, flavanols, and chalcones [10]. They efficiently scavenge $O_{2}^{•-}$, OH, H_2_O_2_, and singlet oxygen and thus, contribute strongly to non-enzymatic antioxidant capacity. Halophytes, in particular, often accumulate high levels of phenolics and flavonoids, which correlate with strong radical-scavenging activity and are proposed to underpin their exceptional stress tolerance [11]. Some inland halophytes frequently show high antioxidant activity and substantial flavonoid content; for example, *Statice gmelinii*, *Artemisia santonicum*, and *Mentha pulegium* are particularly rich in flavonoids [12]. While these correlations strongly implicate flavonoids in halophytic salt adaptation, more work is needed to link specific flavonoid compounds and structural classes to defined tolerance mechanisms.

In many crops, a positive relationship between flavonoid accumulation and tolerance to salinity and other abiotic stress has been reported. This suggests that flavonoids form part of a broader, conserved strategy by which plants buffer oxidative damage, stabilize membranes, and support cellular homeostasis under salt stress. Despite this progress, a unified salinity-focused synthesis of flavonoid function is still lacking. Flavonoid reviews often emphasize UV, drought, temperature, or developmental roles, treating salinity only briefly, whereas salinity reviews typically group flavonoids into a generic “antioxidant” category without resolving which flavonoid branches, structural modifications, or regulatory circuits drive tolerance across tissues and genotypes. Recent studies increasingly show that flavonoids function beyond simple ROS scavenging and are instead integrated into hormonal, ionic, and epigenetic signaling pathways that shape whole-plant salt acclimation. Therefore, framing flavonoids as dynamic network components, rather than endpoint metabolites, is essential for translating mechanistic knowledge into crop improvement.

Here, we present a salinity-centered network-based review of flavonoid biology. Specifically, we aim to (i) summarize the flavonoid biosynthetic framework and how salt stress remodels pathway flux at transcript, enzyme, and metabolite levels; (ii) dissect multi-layer regulatory control under salinity, including transcription factors, hormone integration, ROS feedback loops, and epigenetic/noncoding RNA coordination; (iii) evaluate mechanistic evidence for flavonoid functions in antioxidant defense, ionic/osmotic homeostasis, membrane stabilization, and stress signaling; and (iv) highlight emerging genetic, epigenetic, and breeding strategies that exploit flavonoid-centered networks to engineer or select salt-resilient crops. By integrating these dimensions, we propose a conceptual flavonoid-centered stress network (FCSN), in which distinct flavonoid classes act as nodal integrators linking metabolism, hormones, and signaling. Viewing flavonoids through this network lens provides a basis for more rational deployment of flavonoid pathways in increasingly saline agroecosystems.

## 2. Flavonoid Biosynthesis and Regulation Under Salt Stress

### 2.1. Biosynthetic Pathway Overview

Flavonoids are a large and structurally diverse family of phenolic metabolites ubiquitously distributed across vascular plants. Their biosynthesis branches from the phenylpropanoid pathway. Phenylalanine is first deaminated by phenylalanine ammonia-lyase (PAL); then, it is hydroxylated by cinnamate 4-hydroxylase (C4H), and, finally, it is activated by 4-coumaroyl-CoA ligase (4CL) to generate 4-coumaroyl-CoA, the central C_6_-C_3_ precursor for downstream flavonoid formation. Chalcone synthase (CHS) catalyzes the first committed step of the pathway, condensing one molecule of 4-coumaroyl-CoA with three molecules of malonyl-CoA to form naringenin chalcone. Chalcone isomerase (CHI) rapidly cyclizes this open-chain chalcone to naringenin, thereby establishing the characteristic C_6_-C_3_-C_6_ scaffold [13].

Naringenin thereafter functions as a metabolic hub, from which multiple branches diverge. Flavone synthase (FNS) converts flavanones into flavones; in legumes, isoflavone synthase (IFS) catalyzes the aryl-migration that generates Fabaceae-specific isoflavones, while flavanone 3-hydroxylase (F3H) hydroxylates flavanones at C-3 to yield dihydroflavonols. These intermediates can be further modified: B-ring hydroxylations by flavonoid 3′-hydroxylase (F3′H) and flavonoid 3′,5′-hydroxylase (F3′5′H) yield dihydrokaempferol, dihydroquercetin, and dihydromyricetin, which serve as substrates for downstream flavonol and anthocyanidin pathways [14,15].

From this metabolic node, two dominant branches emerge. First, FLS converts dihydroflavonols to flavonols by introducing a C2-C3 double bond. Second, dihydroflavonol 4-reductase (DFR) and anthocyanidin synthase (ANS) act sequentially to produce leucoanthocyanidins and then anthocyanidins, the core pigments of many flowers, fruits, and stress-responsive tissues [16,17]. In some species, leucoanthocyanidins and anthocyanidins are channeled toward condensed tannins (proanthocyanidins) through reduction by leucoanthocyanidin reductase (LAR) and anthocyanidin reductase (ANR), forming catechin and epicatechin units that subsequently polymerize [18,19]. In the final tailoring steps, UDP-dependent flavonoid glycosyltransferases (UFGTs) conjugate sugars to hydroxyl groups on anthocyanidins, flavanols, and other aglycones, greatly enhancing solubility, stability, subcellular sequestration, and color diversity [20,21]. Additional modification by acyltransferases and O-methyltransferases further expands the structural landscape, creating a flexible flavonoid pool that can be dynamically adjusted in response to salt and other abiotic stresses.

### 2.2. Regulatory Control Under Salinity

#### 2.2.1. Transcription Factors

The flavonoid biosynthetic pathway is controlled by a multilayered transcriptional network in which MYB, bHLH, NAC, and other TF families play central roles. Under salinity, these regulators integrate stress signals with phenylpropanoid and flavonoid pathway activation, often in a species- and branch-specific manner. For example, in mulberry (glycophyte) seedlings, salt stress triggers a selective activation of flavonoid-related transcription factors including MYB123, bHLH42, and NAC factors that correlate with catechin levels, indicating coordinated TF control of specific flavonoid branches under salinity [22]. Multi-omics evidence links this regulatory change with elevated levels of quercetin derivatives, kaempferol, taxifolin, and apigenin [22]. In *Arabidopsis* (glycophyte), overexpression of *AtMYB12* markedly increases flavonoid accumulation and enhances salt tolerance [23]. Recent work in *Ginkgo biloba* (glycophyte) revealed that the lncNAT11-MYB11-F3’H/FLS module drives flavonol biosynthesis under salt stress, and elevated flavonols significantly enhance ROS scavenging and tolerance [24]. In grapevine (glycophyte), the transcription factor VvMYBF1 functions as a positive regulator of flavonoid biosynthesis, enhances flavonoid accumulation, and confers improved salt tolerance [25]. Salt-induced phosphorylation of *GmMYB173* in soybean increases its affinity for the *GmCHS5* promoter, promoting its transcription and the accumulation of dihydroxy B-ring flavonoids with putative roles in salt tolerance [26]. Overexpression of *Scutellaria baicalensis* (glycophyte) *SbMYB2* and *SbMYB7* increased phenylpropanoid accumulation and enhanced NaCl, mannitol, and ABA stress tolerance in transgenic tobacco [27]. In *Erigeron breviscapus*, EbbHLH80 confers improved salt tolerance through elevated flavonoid production and enhanced antioxidant defense [28].

Under salt stress, NAC13-overexpressing poplar plants show enhanced tolerance, whereas NAC13-suppressed lines are hypersensitive [29]. In *G. biloba*, low salt levels stimulate flavonoid biosynthesis alongside NAC transcription factor activation, indicating a coordinated transcriptional response to salinity [30]. NAC transcription factors, classically associated with seed germination, secondary wall formation, and lateral root development, are also increasingly recognized as important regulators of stress-induced flavonoid biosynthesis [31]. Collectively, MYB, bHLH, and NAC TFs activate downstream structural and regulatory genes of the flavonoid pathway and enhance tolerance to salt stress. These examples position flavonoid biosynthesis as a transcriptionally integrated nexus linking developmental regulation with salt-responsive signaling.

#### 2.2.2. Hormonal Integration (ABA, JA, Auxin, Ethylene)

Salt stress triggers a rapid reconfiguration of plant hormone networks, and flavonoid biosynthesis is tightly embedded within these hormonal responses. ABA and JA are typically early responders to osmotic and ionic stress, while auxin and ethylene shape growth–defense trade-offs and tissue-specific acclimation [32].

ABA (Abscisic acid) is a primary driver of flavonoid induction under salinity. Salt-enhanced ABA synthesis activates ABRE-binding transcription factors (AREB/ABF) and downstream MYB, bHLH, and NAC regulators that upregulate phenylpropanoid genes and core flavonoid biosynthetic genes [33,34,35]. In grapevine, *VvbHLH1* overexpression enhances salt and drought tolerance by simultaneously activating ABA-responsive pathways and elevating flavonoid accumulation, providing a clear example of an ABA-linked transcriptional module that couples stress perception to flavonoid production [36]. In strawberry, mild salt stress and exogenous ABA treatments both increase anthocyanins and other phenylpropanoids in parallel with ABA-related gene expression, illustrating this ABA–flavonoid coupling at the whole-organ level [33]. Similar ABA-dependent induction of flavonols and anthocyanins has been reported in rice, where enhanced flavonoid accumulation aligns with improved ROS buffering and maintenance of photosynthetic performance under salt stress [37,38].

Jasmonic acid (JA) provides a second stress-signaling axis that acts in parallel with, and often synergistically to, ABA under salt and osmotic stress. JA/JA-Ile perception via the COI1-JAZ co-receptor releases MYC2 to activate stress and flavonoid pathway genes, and osmotic stress studies show that jasmonates are indispensable for full tolerance, highlighting the JA-JAZ-MYC2 module as a key complement to ABA-driven flavonoid induction [39,40,41]. Across species, JA-responsive MYC2/JAZ modules can co-activate flavonoid biosynthesis together with broader defense programs under salinity [42,43].

Auxin integrates developmental plasticity with flavonoid metabolism under salinity. Salt stress reshapes auxin gradients via changes in biosynthesis, conjugation, and PIN-mediated polar transport, thereby altering root elongation, lateral root formation, and halotropic bending away from saline patches [44,45,46]. Flavonoids, in turn, act as endogenous modulators of auxin transport, locally inhibiting PIN activity and reinforcing new auxin distribution patterns [47,48]. In response to salt stress, the accumulation of key flavonoids activates the transcription of auxin and JA biosynthesis and signaling genes. This hormonal activation, in turn, stimulates cell wall modification and promotes growth, demonstrating that flavonoids function as upstream regulators that initiate protective growth responses under salinity [49].

Ethylene is a key but context-dependent regulator of plant responses to salinity. In alfalfa and *Arabidopsis*, enhanced ethylene signaling can promote salt tolerance by supporting growth and limiting Na^+^ uptake [50,51], whereas, in rice, reduced ethylene sensitivity or disruption of *OsEIL1/2* improves tolerance, indicating that excessive ethylene signaling can be detrimental [52]. Evidence from Japanese apricot shows that ABA–ethylene crosstalk coordinately controls pigment metabolism (chlorophyll degradation and carotenoid accumulation) through ACS/ACO, ERF, and carotenoid biosynthetic genes, illustrating how ethylene signaling under stress is tightly linked to metabolic reprogramming, including pathways for secondary metabolites [53].

### 2.3. Structural Diversification and Functional Relevance Under Salt Stress

The core flavonoid skeleton serves as a template for an extensive array of structural modifications. Among these, glycosylation and methylation are paramount, critically determining the subcellular localization, stability, bioavailability, and specific biological activity of flavonoids, thereby fine-tuning their role in stress adaptation. Recent studies have begun to clarify how these modifications operate within the intricate regulatory networks and physiological responses activated during salt stress.

#### 2.3.1. Methylation

Evidence indicates that O-methylation and specific B-ring substitution patterns significantly enhance flavonoid effectiveness under salt stress. In rice, the cytochrome P450 enzyme CYP75B4 promotes accumulation of the O-methylated flavone tricin, which contributes to both ROS detoxification and reinforced lignin deposition in cell walls. *CYP75B4*-overexpressing plants show reduced ROS, improved Na^+^/K^+^ balance, and stronger salt tolerance, whereas *cyp75b4* mutants are tricin-deficient, lignin-reduced, and salt-sensitive; exogenous tricin partially restores tolerance, confirming its functional role [54].

Halophytes likewise tend to accumulate structurally specialized flavonoids under salinity. In *Sesuvium portulacastrum*, salt stress induces strong accumulation of highly O-methoxylated flavone-7-O-rutinosides together with changes in Na^+^/K^+^ partitioning, elevated H_2_O_2_ in roots, and induction of SOD, CAT, and APX, suggesting that these methoxylated flavonoids contribute to ion homeostasis and oxidative stress management in this halophyte [55]. Similarly, metabolomic profiling of *Salicornia europaea* shows strong induction of quercetin and kaempferol derivatives, with shifts in hydroxylation patterns that reflect selective production of structurally specialized antioxidants optimized for saline environments [56]. In grape suspension cells, NaCl enhances accumulation of proanthocyanidins and anthocyanins lacking C5′-hydroxylation, a modification linked to improved stress acclimation and coordinated cell wall remodeling [57]. Thus, methylation patterns and B-ring substitution help specify which flavonoids are preferentially deployed under salinity.

#### 2.3.2. Glycosylation

Glycosylation is a major axis of structural diversification that shapes the stability, solubility, compartmentation, and signaling capacity of flavonoids and stress-related proteins [58]. At the small-molecule level, most flavonoids occur as O-glycosides formed by UDP-glycosyltransferases (UGTs), a large enzyme superfamily that expanded dramatically during land–plant evolution, including cotton (274) [59], maize (147) [60], soybean (149) [61], and alfalfa [62]. This expansion mirrors the increasing need to fine-tune secondary metabolism and hormone homeostasis as plants adapted to fluctuating terrestrial environments, and many UGTs are now recognized as being responsive to abiotic stresses and are capable of modulating plant stress phenotypes.

Several recent studies directly link flavonoid glycosylation by UGTs to salt tolerance. In rice, overexpression of UGT2 and DUGT2 enhances growth and survival under NaCl, whereas loss-of-function mutants display heightened sensitivity to both salt and drought stress [63]. These phenotypes are accompanied by increased expression of ROS-scavenging and stress-responsive genes, higher accumulation of flavonoid glycosides, and improved antioxidant capacity, whereas *dugt2* mutants show the largest drop in flavonoid content and antioxidant gene expression, operating under the upstream control of bZIP23 and bZIP16, respectively [63,64]. Similar patterns are seen in other species: overexpression of UGT79B2/UGT79B3 in *Arabidopsis* enhances tolerance to salt, drought, and low temperature by modulating anthocyanin metabolism [65]; CrUGT87A1 from *Carex rigescens* confers higher germination, better growth, and elevated flavonoid levels in transgenic *Arabidopsis* under high salinity [66]; and UGT71C4 in cotton influences embryo proliferation and seed development [67]. Collectively, these data support a model in which flavonoid-directed UGTs canalize carbon into stable glycosides that act as ROS buffers, membrane protectants, and signaling mediators during salt stress.

Other UGTs primarily affect osmotic adjustment and primary metabolism but still contribute to salt acclimation. The salt-inducible Arabidopsis gene UGT85A5 enhances NaCl tolerance when expressed in tobacco, with higher germination, better growth, more proline and soluble sugars, and a lower MDA and Na^+^/K^+^ ratio, alongside induction of sucrose, hexose transport, and LEA genes [68]. These observations suggest that glycosylation not only shapes flavonoid pools, but also stabilizes primary metabolic and osmoprotective networks during salinity. Beyond small molecules, protein N-glycosylation provides a second layer of glycan-based stress adaptation. The hexosamine biosynthetic pathway generates UDP-GlcNAc, the donor for N-glycans, via GlcNAc1pUTs (GlcNA.UTs) [69]. Interference with Arabidopsis mutants disrupts N-glycosylation, triggers the unfolded protein response, and causes strong salt hypersensitivity, and this is alleviated by blocking ABA biosynthesis [70]. Likewise, defects in N-glycan maturation in cgl1 and stt3a mutants lead to growth inhibition, abnormal root tips, and callose accumulation under salinity. In both cases, mis-glycosylation of the plasma membrane glycoprotein KOR1/RSW2, essential for cellulose biosynthesis, compromises cell wall integrity, and cellulose-deficient rsw1-1 and rsw2-1 lines are similarly salt-sensitive [71,72]. N-glycoproteomic studies in tomato and sorghum further show that salinity remodels N-glycosylation of proteins involved in secondary metabolism, amino acid biosynthesis, cell wall organization, and energy production, linking N-glycan structure to salt stress resilience [73,74]. Overall, glycosylation of both metabolites and proteins contributes to maintaining redox balance, membrane and cell wall integrity, and osmotic adjustment under salt stress.

## 3. Functional Roles of Flavonoids in Salt Stress Tolerance

**Figure 1:**
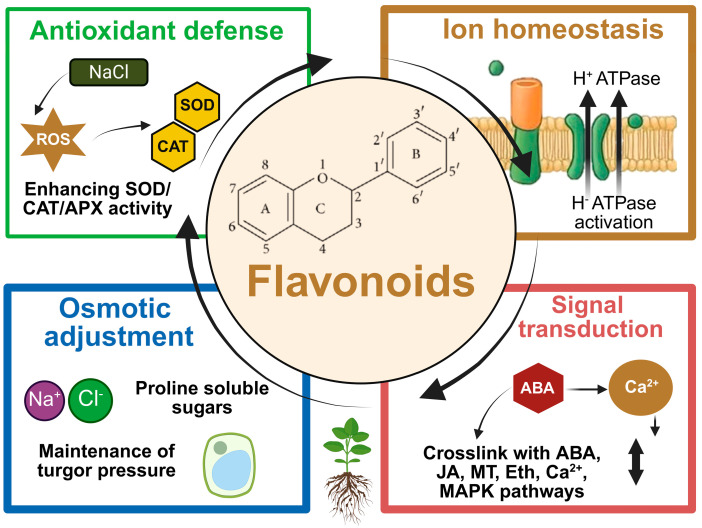
Multifunctional roles of flavonoids in plant salt stress adaptation. Flavonoids support antioxidant defense, osmotic adjustment, ion homeostasis, and signaling integration under salinity.

Experimental and field evidence now indicate that flavonoids are active mediators of salt stress tolerance rather than passive stress markers. Acting at multiple levels, they integrate antioxidant defense, ion and osmotic homeostasis, membrane protection, and signaling crosstalk within the salt stress network. As summarized in Figure 1, flavonoids synergize antioxidant defense with ionic homeostasis and stress signaling, thereby supporting whole-plant acclimation under salinity. These functions arise from their tight embedding in transcriptional, hormonal, and epigenetic regulatory circuits. In the following subsections, we summarize how flavonoids contribute to cellular protection, ion balance, and signal integration under salinity.

### 3.1. Antioxidant and Redox Buffering Systems

Salinity-induced oxidative stress results from the overproduction of reactive oxygen species (ROS), which can cause significant damage to cellular components when they exceed the capacity of enzymatic antioxidant defenses. Flavonoids function as crucial non-enzymatic antioxidants, providing a complementary redox buffer to mitigate this damage [75]. Their chemical structure, characterized by multiple hydroxyl groups and conjugated rings, allows them to effectively quench free radicals and inhibit ROS-generating enzymes. Flavonoids accumulate in subcellular compartments such as chloroplasts and vacuoles, where they can quench singlet oxygen and hydrogen peroxide, a mechanism widely proposed as a first-line antioxidant defense under oxidative or environmental stress. Because salinity strongly promotes ROS production and oxidative injury [76], such compartmentalized flavonoid deployment may provide critical protection under salt stress.

Flavonoids are well-established antioxidants in higher plants [77,78]. Subcellular localization reinforces this role: large pools of flavonoids accumulate in mesophyll vacuoles and chloroplasts. Chloroplast-associated flavonoids effectively quench ^1^O_2_ generated under excess blue light [79], while vacuolar flavonoids in mesophyll and epidermal cells scavenge diffusing H_2_O_2_, buffering oxidative stress [78]. Flavonoids function as major non-enzymatic antioxidants in plants, especially when they accumulate in chloroplasts and vacuoles. These compartmentalized pools can quench singlet oxygen and other ROS and contribute increasingly to redox protection under strong photo-oxidative or salinity stress [77]. Vacuolar sequestration is mediated by ABC transporters, GST-associated transport, and vesicle trafficking, enabling the safe accumulation of large antioxidant flavonoid reservoirs during stress [80,81].

Salt stress often induces a targeted reconfiguration of the flavonoid pathway towards more potent antioxidants. In *G. biloba*, *GbTOE1a* overexpression elevates flavonoid accumulation and enhances antioxidant capacity under NaCl, whereas silencing reduces tolerance [82]. Glutathione S-transferase (GST) activity, involved in vacuolar sequestration of flavonoids, is also enhanced [83]. In *Myrtus communis* and *Pistacia lentiscus*, carbon allocation to myricetin and quercetin glycosides is increased under salinity, especially in the more sensitive *M. communis*, where these flavonols accumulate in palisade cells and likely participate in peroxidase-mediated H_2_O_2_ reduction [84]. Root zone salinity and UV radiation induce similar patterns, such as the strong upregulation of quercetin 3-O-glycosides and luteolin 7-O-glycosides, while mono-hydroxylated B-ring flavones remain largely unaffected, indicating a shift towards more efficient antioxidant structures [78,85].

This biochemical role is strongly supported by genetic evidence. In *Arabidopsis*, overexpression of *UGT76E11* or the transcription factor MYB112 enhances flavonoid accumulation, leading to reduced ROS levels and improved salt tolerance [86,87]. Similarly, the heterologous expression of the grape bHLH factor *VvbHLH1* in *Arabidopsis* elevates flavonoid levels and upregulates ABA biosynthetic genes, enhancing tolerance to salt and drought [36]. Beyond transcriptional control, epigenetic regulation also fine-tunes this antioxidant system; for example, overexpression of the *Arabidopsis* DNA demethylase *AtROS1* in tobacco promotes the demethylation and activation of key flavonoid biosynthetic genes (*CHS*, *CHI*, *F3H*, *FLS*), resulting in higher flavonoid accumulation and improved salt tolerance [88]. In *Solanum nigrum*, NaCl treatment upregulates *SnPAL*, *SnCHS*, and *SnFLS*, leading to large increases in quercetin-3-β-D-glucoside, which correlates tightly with enhanced antioxidant capacity under salt stress [89]. Overexpression of *CHS* genes in *Eupatorium adenophorum* and *Abelmoschus esculentus* raises flavonoid levels and improves salt tolerance by maintaining ROS homeostasis [90]. In tobacco, *NtCHS1* overexpression upregulates *4CL*, *CHI*, *DFR*, and *ANS*, increases rutin accumulation, lowers $O_{2}^{•-}$ and H_2_O_2_, and confers greater salinity tolerance [91]. Together, these biochemical, physiological, and genetic data indicate that flavonoids form a flexible redox buffer that complements enzymatic defenses and limits oxidative damage under salinity. Importantly, reducing ROS pressure also limits lipid peroxidation, which helps preserve membrane integrity and the activity of membrane-bound ion transport systems; thus, flavonoid-mediated redox buffering can translate into improved Na^+^/K^+^ homeostasis under salinity.

### 3.2. Ionic and Osmotic Homeostasis

Salt stress perturbs ion balance by elevating cytosolic Na^+^ and competing with K^+^ at transporter- and enzyme-binding sites, disrupting membrane potential, enzyme activity, and osmotic regulation. To counter this, plants combine antioxidant defenses with mechanisms that restrict Na^+^ uptake, promote Na^+^ extrusion or vacuolar sequestration, and maintain a high cytosolic K^+^/Na^+^ ratio. Among non-enzymatic antioxidants, flavonoids, together with glutathione, other phenolics, carotenoids, and compatible solutes, play a central role in protecting ion transport machinery, buffering ROS around membranes and transporters and thereby supporting ionic and osmotic homeostasis under salinity [92].

Flavonoids contribute to ionic and osmotic homeostasis primarily by protecting ion transport systems from ROS-induced dysfunction and, in some cases, by modulating the activity of specific Na^+^ and K^+^ transporters. In quinoa, the strong induction of the flavonol rutin (up to 25-fold under salinity) enhances tissue tolerance by sustaining K^+^ retention and Na^+^ efflux, thereby preserving photosynthetic performance under high salt [93]. Central to Na^+^ detoxification is vacuolar Na^+^/H^+^ antiporters (NHX family) and the SOS signaling pathway, which jointly mediate Na^+^ extrusion and compartmentation. For example, overexpression of AtNHX1 in kiwifruit increased vacuolar Na^+^ sequestration, improved osmotic adjustment, and strengthened antioxidative capacity, leading to higher salt tolerance [94]. Maintaining high cytosolic K^+^ and low Na^+^ is essential for enzyme stability and membrane potential; however, excess Na^+^ competitively inhibits K^+^ uptake through channels such as AKT1, leading to ionic disequilibrium [95].

In rice, enhanced accumulation of flavonoids such as kaempferol and quercetin integrates into the ion homeostasis network by scavenging ROS and reprogramming ion transport: F3H overexpression upregulates HKT and SOS genes, downregulates NHX, and helps maintain a favorable Na^+^/K^+^ balance under salt [96]. The SOS3-SOS2-SOS1 signaling module, which extrudes Na^+^ or promotes vacuolar sequestration, also interfaces with anthocyanin regulation: SOS3 signaling activates AIR1, a bZIP transcription factor that controls anthocyanin accumulation under salt stress by inducing *F3H*, *F3′H*, and *LDOX* [97]. This links Ca^2+^-dependent Na^+^ signaling with flavonoid biosynthesis so that restoration of ion homeostasis is accompanied by reinforcement of antioxidant capacity. Anthocyanins also intersect with SA and ABA signaling. By modulating ROS, they influence SA-dependent pathways [98], and SA/ABA can enhance late anthocyanin biosynthetic genes (*VcDFR1*, *VcA3GT2*) via the MYB factor VcMYBA, improving oxidative stress tolerance and salt resilience [99]. These observations indicate that flavonoids support Na^+^/K^+^ homeostasis indirectly by stabilizing ion transport systems and integrating with SOS- and hormone-dependent signaling under salinity.

### 3.3. Membrane Stabilization and Osmoprotection

Cellular membranes are primary targets of salt-induced damage. ROS and ionic toxicity drive lipid peroxidation, alter bilayer fluidity and permeability, and impair membrane-bound proteins and transporters [100]. Flavonoids act as membrane-active polyphenols that mitigate these effects. Their amphipathic structures enable them to insert into lipid bilayers, where they interact with phospholipid headgroups and acyl chains, modulating membrane fluidity, order, and leakiness [101]. The magnitude and direction of these effects depend strongly on structural features (hydroxylation, methoxylation, glycosylation), making structure–activity relationships critical for biological activity [102].

This membrane-stabilizing function provides a mechanistic bridge between antioxidant activity and ion homeostasis under salt stress. Lipid peroxidation and increased membrane leakiness promote electrolyte leakage and K^+^ loss and can compromise the function of membrane-embedded transporters required for Na^+^ exclusion and/or vacuolar sequestration. By suppressing peroxidation-driven membrane damage and stabilizing bilayer properties, flavonoids help maintain transporter integrity and selectivity, thereby supporting Na^+^/K^+^ balance in salt-exposed plants.

Under abiotic stress, increased flavonoid accumulation often coincides with improved membrane integrity. In grape leaves, stress-induced flavonoids (e.g., anthocyanins) are associated with enhanced tolerance, most likely through reduced lipid peroxidation and preservation of bilayer structure [103]. Although these studies are not all conducted under salinity, they provide a mechanistic basis for similar roles during salt stress. Notably, salt stress couples oxidative injury with transporter dysfunction; therefore, any flavonoid-driven reduction in peroxidation is expected to stabilize membrane potential and preserve transporter activity indirectly through improved bilayer properties.

Under saline conditions, flavonoids act together with compatible solutes (proline, soluble sugars, glycine betaine) to stabilize membranes, reduce MDA accumulation, and help maintain osmotic balance. The accumulation of specific highly methoxylated flavone glycosides in the halophyte *Sesuvium portulacastrum* under salinity is a prime example of such a specialized membrane-protective adaptation [104]. Overexpression of *MsPAL1* in alfalfa increases flavonoid and lignin biosynthesis, boosts ROS scavenging, and confers strong improvement in salt tolerance [105]. Thus, in addition to their chemical antioxidant role, flavonoids exert a physical protective function at the membrane level, adjusting bilayer properties and preserving the activity of embedded proteins, an essential component of osmoprotection during salt stress. Together, these membrane-level effects provide a mechanistic explanation for why flavonoid accumulation frequently co-occurs with improved Na^+^/K^+^ balance and reduced stress injury in salt-exposed plants.

To consolidate species-level evidence across model and crop systems, Table 1 summarizes experimental studies linking flavonoid metabolism with salt stress phenotypes.

### 3.4. Interaction with Stress Signaling Pathways

#### 3.4.1. Flavonoid Integration into Ca^2+^, ABA, MAPK, and Melatonin Signaling Under Salt Stress

Salinity rapidly alters cytosolic Ca^2+^ levels, and Ca^2+^ acts as a central second messenger that coordinates ion toxicity, osmotic stress, and hormone signals. Ca^2+^ not only stabilizes membranes and reduces ion leakage, but also promotes osmolyte accumulation (proline, glycine betaine), contributing to osmotic adjustment and structural protection. Ca^2+^ signatures are decoded by sensors such as calmodulin (CaM), calmodulin-like proteins (CMLs), calcineurin B-like proteins (CBLs), and Ca^2+^-dependent protein kinases (CDPKs/CPKs), which relay information to hormonal and transcriptional networks [106,107]. Exogenous Ca^2+^ can stimulate phenylpropanoid and flavonoid pathways. In fresh-cut *Cucumis melo* (cantaloupe), CaCl_2_ treatment upregulates *PAL*, *C4H*, and *4CL* and induces the expression of *CmPALs*, thereby increasing total phenolics and activating the phenylpropanoid pathway [108]. Under salinity, calcium phosphate nanoparticles (CaP-NPs) elevate flavonoids and antioxidant enzymes while reducing H_2_O_2_ [109]. In buckwheat sprouts, CaCl_2_ modifies C-glycosylflavones (vitexin, orientin, isovitexin) and rutin levels [110]. In *Gleditsia sinensis*, CaCl_2_ elevates L-phenylalanine, kaempferol, ferulic acid, and catechin under salinity, indicating that Ca^2+^ signaling can modulate flavonoid profiles during salt stress [111]. These Ca^2+^-dependent changes intersect with hormone networks, particularly ABA. CDPKs activated by Ca^2+^ can stimulate ABA-responsive elements involved in stomatal closure and ion homeostasis [112], helping adjust Ca^2+^ flux and water loss under salinity.

Ca^2+^-independent pathways, especially MAPK cascades, also participate in salt and flavonoid signaling. MAPK modules (MAPKKK-MAPKK-MAPK) transduce and amplify stress signals to transcriptional and metabolic targets [113]. In soybean, heterotrimeric G-proteins activate phospholipase-linked GMK1 for early stress signaling [114]. These cascades intersect with hormone and flavonoid regulators, although direct links to specific flavonoid branches under salt stress remain to be fully elucidated. Melatonin (MT) is another key node that connects redox, hormone, and flavonoid pathways. MT mitigates salt-induced H_2_O_2_ accumulation, promotes Na^+^/K^+^ pumping, stimulates detoxification enzymes, and enhances flavonoid accumulation. MT treatment in grape berries (*Vitis vinifera*) increases total phenolics, flavonoids, and DPPH scavenging capacity and upregulates *PAL*, *STS*, *4CL*, and *CHS*, enhancing the accumulation of flavanones, flavanols, and other flavonoids [115]. MT also enhances CHS activity under stress in tomato, promoting anthocyanin production [116]. In *Brassica juncea*, MT combined with *Pseudomonas fluorescens* elevates kaempferol, cyanidin, naringenin, quercetin, and myricetin [117]. Together, Ca^2+^-dependent CDPKs, MAPK cascades, ABA, and MT converge on phenylpropanoid–flavonoid metabolism and redox control so that flavonoids act both as downstream products and feedback regulators within this shared signaling network under salt stress.

#### 3.4.2. Epigenetic Influence and Noncoding RNAs

Epigenetic regulation adds a higher-order control layer to salt stress signaling, shaping how flavonoid-centered responses are deployed. Under salinity, DNA methylation and histone marks are dynamically remodeled at transporters, hormone-related genes, and phenylpropanoid/flavonoid enzymes, thereby tuning the sensitivity of ROS and ABA/JA pathways. For example, demethylation by the DNA glycosylase AtROS1 activates flavonoid biosynthetic and antioxidant genes under salt stress, enhancing ROS detoxification and tolerance [88]. Salt-primed soybean seedlings similarly show shifts in H3K4me2/H3K4me3 and H3K9ac at stress loci, consistent with a chromatin-based “memory” of salinity [118]. RNA-directed DNA methylation (RdDM) and noncoding RNAs (ncRNAs) couple these epigenetic changes to stress signaling. In *Arabidopsis*, salt-induced alterations in 24-nt siRNAs and CHH methylation at the AtMYB74 promoter derepress this MYB, which then activates ABA/ROS-responsive genes such as RD29B, RAB18, and RD20 [119]. Recurrent osmotic/salt stress generates differentially methylated regions (DMRs) that are inherited via the maternal germline and improve progeny performance; at the CNI1 locus, a stress-induced antisense lncRNA (CNI1-AS1) and a hyperosmotic DMR jointly modulate CNI1 expression [120]. These cases illustrate how RdDM, DMRs, and ncRNAs gate the expression of E3 ligases, transcription factors, and transporters that interface with flavonoid metabolism, hormone signaling, and ion homeostasis.

lncRNAs in crop species extend this framework and directly affect classical salt tolerance traits. In cotton, lncRNA973 is strongly induced by NaCl; its overexpression enhances salt tolerance, whereas silencing causes wilting, oxidative damage, and Na^+^/K^+^ imbalance [121]. lncRNA973 regulates ROS-scavenging genes (SOD, CAT, POD), ion transporters (e.g., NHX7), osmolyte biosynthesis (P5CS), and TFs such as MYB5, WRKY46, NAC29, and ERF62 and may interact with the miR399-PHO2 module [121]. In sweet sorghum, lncRNA13472 likely acts as a competing endogenous RNA for sbi-MIR169b, influencing a proton pump subunit and salt tolerance [122]. In *M. truncatula*, MtCIR2 functions as a salt-induced lncRNA that increases expression of the ABA catabolic gene *CYP707A2* and suppresses GA20-oxidases, thereby sensitizing seed germination to salinity [123].

**Figure 2:**
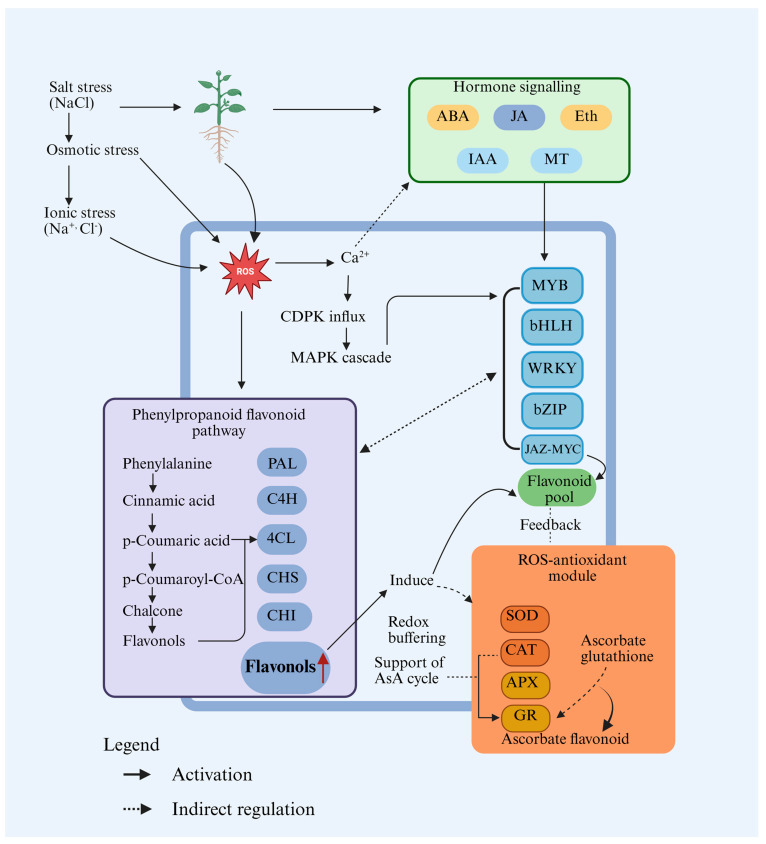
Flavonoids at the cellular core of the salt stress network. Salt-induced ROS and Ca^2+^ influx, together with ABA, JA, ethylene, and auxin signaling, activate transcription factor modules and the phenylpropanoid-flavonoid pathway, which, in turn, enhances antioxidant enzymes and feeds back to redox and signaling circuits.

Together, these examples indicate that DNA methylation, histone modifications, siRNAs, and lncRNAs determine when and where flavonoid-based defenses are deployed during salt stress. Epigenetic regulation and ncRNAs act through shared regulatory nodes such as MYB/NAC/WRKY transcription factors, antioxidant enzymes, and ion transporters that control flavonoid biosynthesis and ROS detoxification. By tuning these nodes, epigenetic mechanisms position flavonoids as central metabolites linking ROS signaling, hormone responses, and ion balance during salinity stress. These connections are summarized in Figure 2.

To elucidate “how” flavonoids confer tolerance, Table 2 organizes the major functional categories and representative modules through which flavonoids modulate salt stress responses.

## 4. Spatial and Inter-Organ Roles of Flavonoids in Salt Stress

In addition to directly alleviating ROS through electron transfer, flavonoids can remodel the antioxidant proteome via post-translational regulation and act as metabolic modulators in stress signaling networks. Owing to their structural heterogeneity and tissue-specific regulation, flavonoids support a broad range of physiological functions and contribute to flexible organ-specific responses to environmental stress.

### 4.1. Root–Shoot Communication and Auxin-Mediated Morphogenesis Under Salt Stress

**Figure 3:**
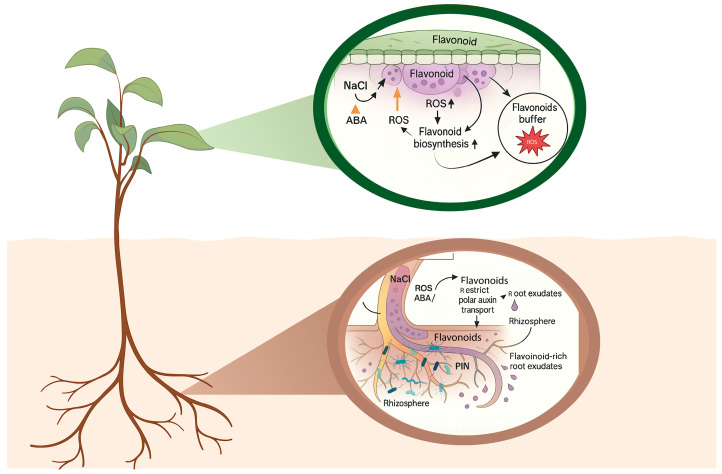
Spatial deployment of flavonoids in leaves and roots under salinity. Leaves accumulate flavonoids in epidermal and mesophyll tissues to buffer ROS and protect membranes, whereas roots adjust auxin transport and secrete flavonoid-rich exudates that condition the rhizosphere.

Localized environmental constraints such as soil salinity require controlled long-distance transport of stress signals to coordinate acclimation at the whole-plant level [124]. Flavonoids are key components of this systemic communication. By modulating polar auxin transport, they influence root formation and elongation, as well as phototropic and gravitropic responses [48]. In legumes, flavonoids function as essential signals for the initiation of Rhizobium–legume symbiosis and nodule development and can serve as a carbon source for rhizosphere microorganisms with appropriate catabolic enzymes [125]. Accumulated isoflavonoids in alfalfa have been proposed to prevent early symbiosis inhibition under salinity by sustaining nod gene expression and promoting localized auxin accumulation required for nodulation [126]. Flavonoid-dependent tuning of auxin and ABA signaling in roots and shoots contributes to these systemic architectural responses [43,127]. Mechanistically, long-distance mobility is most plausible for more water-soluble flavonoid conjugates (especially O-glycosides and acyl-glycosides), whereas aglycones are less stable and less compatible with aqueous vascular streams. Recent advances in flavonoid transport research highlight that glycosylation and further decoration steps are tightly coupled to intracellular trafficking and long-distance distribution potential, supporting the concept that “mobile flavonoid signals” are often transported as conjugated forms rather than free aglycones [128,129]. Together, these findings suggest that flavonoids and flavonoid-regulated hormones act as mobile components of root–shoot communication, linking local salt perception in roots to coordinated growth and stress responses in aerial organs. These spatial deployment patterns are outlined in Figure 3.

### 4.2. Apoplastic and Symplastic Distribution Under Stress Conditions

At the tissue and cellular scales, the spatial distribution of flavonoids is a key determinant of their protective function. The accumulation of flavonoids in specialized cells or subcellular locations, including cell walls, membranes, and glandular trichomes, is a prevalent strategy to mitigate oxidative damage under abiotic stress [130,131]. Flavonoids are often enriched in epidermal cells and trichomes, where they act as UV screens and ROS buffers to protect underlying tissues from excess light and oxidative injury. In addition, flavonoids can interact with DNA and help limit oxidative damage at the genomic level [132].

Anthocyanins mitigate salt stress by stimulating osmotic adjustment through the accumulation of proline, glycine betaine, and soluble sugars, in addition to preserving ion homeostasis via the regulation of ion transporters and channels. They influence the expression and activity of Na^+^/H^+^ antiporters and help sustain K^+^/Na^+^ balance by enhancing K^+^ uptake via specific channels and transporters [133]. In Paulownia spp., anthocyanin accumulation protects the photosynthetic apparatus by restricting electron flow from QA to the plastoquinone pool and limiting the photochemical inhibition of both photosystems during short NaCl exposure [134]. Genome-scale evidence in *Rhus chinensis* shows that glandular trichomes are metabolic sites for tannins/flavonoids and contribute to salt-associated ion/metabolite handling [131]. Exogenous naringenin applied to the roots of common bean (*Phaseolus vulgaris*) alleviates salt stress by modulating nitrogen metabolism, cellular redox balance, ROS scavenging, and photosynthetic efficiency [135].

Rutin provides further evidence for compartment-specific action: in quinoa and broad bean, rutin improves K^+^ retention and Na^+^ efflux. The lack of a link between rutin-induced adjustments in K^+^ and H^+^ fluxes indicates that cytosolic rutin mitigates hydroxyl radicals generated under salinity, consequently inhibiting K^+^ loss via ROS-triggered efflux pathways instead of directly regulating voltage-controlled K^+^ channels [93]. In maize, exogenous rutin strengthened the antioxidant system by reducing ROS accumulation (lower TBARS and H_2_O_2_) and improved chlorophyll content, osmolyte accumulation, and leaf relative water content under salt stress [136].

### 4.3. Flavonoids as Root Exudates and Rhizosphere Microbiota

During growth, roots exude a broad array of primary and secondary metabolites that shape the surrounding microbiome. Under salt stress or changing rhizosphere conditions, root tissues can rapidly synthesize and release large amounts of flavonoids and other phytochemicals. Flavonoid aglycones and glycosides in exudates act as communication signals to recruit beneficial rhizosphere microbiota and to modulate soil microbial communities during salt stress [48]. They play central roles in plant–microbe interactions, particularly arbuscular mycorrhizal (AM) symbioses and rhizobial associations [125,137]. Mycorrhiza helper bacteria can produce Nod-like factors and induce root exudation of flavonoids that attract AM fungi and facilitate root colonization [48].

The establishment and activity of rhizobial symbiosis are highly sensitive to salinity, which reduces the formation of deformed root hairs and limits infection sites [125]. Flavonoids strengthen symbiotic signaling in alfalfa and other legumes, and rhizobial inoculation has been associated with improved salt tolerance in certain species [126]. In wheat, inoculation with salt-tolerant bacterial isolates increased rhizosphere phenolics (gallic, caffeic, syringic, vanillic, ferulic, and cinnamic acids) and the flavonoid quercetin; this accumulation correlated with enhanced plant growth promotion under saline conditions [138]. Flavonoid–microbiome interactions represent an efficient means for plants to communicate with and recruit beneficial plant-growth-promoting (PGP) bacteria and fungi, thereby extending flavonoid-mediated salt tolerance beyond the plant body into the rhizosphere.

### 4.4. Facilitation of Systemic Salt Tolerance

Flavonoids are not only ROS scavengers, but they may also contribute to systemic salt acclimation by acting as inter-organ metabolites, typically in conjugated forms (glycosides/acyl-glycosides) that are compatible with long-distance transport. Importantly, increasing evidence supports that flavonoids can occur in vascular sap. For example, metabolomic profiling of citrus phloem sap detected abundant secondary metabolites, with flavonoids representing a major component, including significant detection of flavanone glycosides such as naringin, hesperidin, and neohesperidin, providing direct support that flavonoid conjugates can be present within the phloem stream [139]. In parallel, recent transport-focused syntheses emphasize that glycosylation/acylation enhances solubility and trafficking competence and that long-distance movement likely involves conjugated flavonoids combined with cell-to-cell and vascular-associated transport processes rather than diffusion of aglycones alone [128,129]. They modulate hormonal signaling pathways involving auxin (IAA), ABA, SA, and JA, thereby integrating redox status with growth and defense at both local and distant sites [43,140]. Emerging evidence indicates that specialized metabolites such as flavonoids and glucosinolates do not merely detoxify ROS; they also remodel the antioxidant proteome and post-translational landscape. By acting as metabolic rheostats, they fine-tune MAPK phosphorylation and reshape cross-talk between ABA and jasmonate pathways, establishing coordinated defense programs under salinity [141].

At the gene level, salt stress upregulates flavonoid hydroxylase (*F3H*) in tobacco, promoting flavanol accumulation and enhancing antioxidant enzyme activity [35]. In rice, transcriptome analysis of *OsWNK9*-overexpressing lines under salinity revealed enrichment of metabolic pathways, including phenylpropanoid and other secondary-metabolite biosynthetic routes associated with stress acclimation [142]. In strawberry, signals that induce phenylpropanoid and flavonoid biosynthesis also provoke increases in ABA, and mild salt stress elevates phenylpropanoid content alongside upregulation of flavonoid- and ABA-related genes [33,34]. In *G. biloba*, salt-induced flavonoid accumulation is associated with JA biosynthesis, suggesting a synergistic mechanism in which jasmonate and flavonol metabolism jointly enhance salt tolerance [143]. In *Arabidopsis*, flavonoid biosynthesis is tightly linked to IAA- and JA-signaling genes, which are more strongly induced in transgenic plants than in the wild type under salt stress; IAA and JA levels increase, likely activating cell wall biosynthesis or modification genes, resulting in longer roots and larger shoots under salinity [49]. Similarly, in alfalfa roots, early NaCl exposure co-regulates JA signaling and flavonoid biosynthetic genes, supporting hormone–metabolite coordination at the whole-plant level [43].

Key enzymes reinforce this systemic role. Flavonoid-3′-hydroxylase (*F3′H*), a cytochrome P450 monooxygenase, enhances salt tolerance in soybean by increasing ascorbic acid content, thereby preserving membrane integrity and cellular homeostasis [132]. In rice, salt-induced ABA and SA accumulation regulate primary flavonoid pathway enzymes, leading to elevated kaempferol and quercetin and improved salt resistance [96]. Exogenous ABA further promotes leaf growth and development in rice under stress by modulating flavonoid biosynthesis and linoleic acid metabolism [37]. These findings support a model in which flavonoids, distributed across tissues and compartments, function as both local buffers and systemic modulators of ROS and hormone signaling. Through their combined roles in root exudation, root–shoot communication, apoplastic/vacuolar partitioning, and hormone interplay, flavonoids facilitate long-distance salt tolerance at the whole-plant level.

**Table 1:** Species-based summary of experimental evidence linking flavonoid metabolism to salt stress responses in model and crop plants.

Plant Species/System	Key Flavonoid Module (Genes/Metabolites)	Experimental Evidence Under Salt Stress	Proposed Mechanism of Action	Ref. No.
*Arabidopsis thaliana*	*AtMYB12* overexpression upregulates the expression of *CHS*, *CHI*, *F3H*, *FLS*, *DFR*, and *ANS*	Transgenic *AtMYB12*-OE plants exposed to salinity show increased flavonoid accumulation and improved growth.	Enhanced flavonoid biosynthesis strengthens ROS scavenging and ABA-linked stress signaling, contributing to higher salt tolerance.	[23]
*Ginkgo biloba*	LncNAT11-MYB11-F3’H/FLS module flavonol biosynthesis	Salt-treated seedlings and transgenic analyses show that the lncNAT11-MYB11-F3’H/FLS module drives flavonol accumulation and improves NaCl tolerance.	Elevated flavonols strengthen ROS scavenging and membrane protection, reducing oxidative damage under salinity.	[24]
*Glycine max*	GmMYB173 phosphorylation and activation	Quantitative phosphoproteomics and metabolomics under NaCl reveal that phosphorylated GmMYB173 enhances *CHS* expression and dihydroxy B-ring flavonoid accumulation.	Optimized flavonoid metabolism reinforces ROS buffering and supports ion and osmotic adjustment during salt stress.	[26]
*Arabidopsis thaliana*	UGT79B2 / UGT79B3 (anthocyanin UGTs)	UGT79B2/B3-overexpressing lines exhibit increased anthocyanin accumulation and improved tolerance to salt.	Anthocyanin glycosides act as efficient ROS buffers and membrane protectants, enhancing multi-stress resilience.	[65]
*Ginkgo biloba*	*GbTOE1a* overexpression	*GbTOE1a*-OE plants accumulate more flavonoids and display higher antioxidant capacity and salt tolerance; silencing reduces both.	AP2/ERF TF GbTOE1a promotes flavonoid pathway activation, improving redox homeostasis and stress resilience.	[82]
*Solanum nigrum*	Quercetin-3-β-D-glucoside accumulation	NaCl treatment upregulates *SnPAL*, *SnCHS* and *SnFLS* and causes marked increases in quercetin-3-β-D-glucoside correlated with antioxidant capacity.	Flavonol glycosides contribute to ROS scavenging and protection of membranes and photosynthetic apparatus under salinity.	[89]
*Abelmoschus esculentus*	*CHS* overexpression	Overexpression of *CHS* genes increases flavonoid content in transgenic plants and improves salt tolerance with lower ROS levels.	Upregulated *CHS* diverts carbon into antioxidant flavonoids, enhancing ROS homeostasis and stress protection.	[90]
*Nicotiana tabacum*	*NtCHS1* overexpression elevated rutin	*NtCHS1*-OE tobacco plants accumulate more rutin, show reduced H_2_O_2_ and $O_{2}^{•-}$, and survive better under saline conditions.	Rutin-rich tissues exhibit stronger redox buffering capacity, preserving membrane integrity and photosynthesis under salt stress.	[91]
*Chenopodium quinoa*	Induction of flavonol rutin under NaCl	Salinity strongly induces rutin, coinciding with improved K^+^ retention, Na^+^ efflux, and maintenance of photosynthetic performance.	Rutin supports tissue tolerance by protecting ion transport systems from ROS and sustaining favorable K^+^/Na^+^ balance.	[93]
*Medicago sativa*	*MsEOBI-MsPAL1* module and flavonoids	Overexpression of *MsPAL1* in alfalfa increases flavonoid and lignin levels and confers higher tolerance to salinity accompanied by reduced ROS.	Enhanced phenylpropanoid flux strengthens antioxidant capacity and cell wall reinforcement, improving salt stress tolerance.	[105]
*Euphorbia kansui*	*EkFLS* overexpression	*EkFLS* expression enhances flavonol accumulation and improves growth and survival of plants exposed to NaCl.	Flavonols act as ROS buffers and contribute to osmotic and membrane protection under saline conditions.	[144]
*Medicago truncatula*	MtBGLU17 (β-glucosidase) and antioxidant flavonoids	Loss- and gain-of-function analyses demonstrate that MtBGLU17 promotes accumulation of flavonoid derivatives and enhances salt tolerance.	Hydrolysis and remodeling of flavonoid conjugates fine-tune ROS detoxification and cellular protection under salinity.	[145]

**Table 2:** Functional categories of flavonoid action in plant salt stress responses with representative modules and supporting evidence.

Functional Category	Representative Flavonoid Module(s)	Experimental Systems/Conditions	Mechanistic Summary Under Salinity	Ref. No.
Antioxidant and redox buffering	AtMYB12, MYB112, and UGT76E11; LncNAT11-MYB11-*F3’H*/*FLS*; and *CHS*- and *FLS*-based modules	*Arabidopsis* TF and UGT over expressors; *G. biloba* seedlings and TF lines; tobacco *CHS*/*FLS* overexpression; and *S. nigrum* and quinoa under NaCl.	Flavonols and anthocyanins and their glycosides accumulate in chloroplasts and vacuoles, quenching ROS (H_2_O_2_, $O_{2}^{•-},$^1^O_2_), lowering MDA, and sustaining photosynthetic activity and growth.	[23,24,86,87,89,90,91]
Na^+^/K^+^ and ionic/osmotic homeostasis	Rutin-enriched flavonol pools; *F3H*-dependent anthocyanin pathway; and SOS3-AIR1-anthocyanin module	Quinoa exposed to NaCl; *F3H*-OE rice lines; and mutants and over expressors affecting SOS3–AIR1 signaling and anthocyanin biosynthesis.	Flavonoids protect plasma membrane H^+^-ATPases and Na^+^/K^+^ transporters from oxidative damage and, in some cases, transcriptionally reprogram HKT, SOS, and NHX genes, supporting K^+^ retention, Na^+^ efflux, and vacuolar sequestration.	[93,94,96,97]
Membrane stabilization and osmoprotection	Highly O-methoxylated flavone-7-O-rutinosides; *MsPAL1*-driven phenylpropanoids; and flavonol glycosides	Halophyte *Sesuvium portulacastrum* under high salinity; alfalfa *MsPAL1* overexpression lines; and diverse species with salt-induced flavonol glycosides.	Amphipathic flavonoids insert into lipid bilayers, limit peroxidation, adjust fluidity and leakiness, and act together with compatible solutes (proline, sugars) to maintain membrane integrity and cellular hydration.	[55,84,95,104,105]
Hormonal and Ca^2+^/MAPK signaling integration	Flavonoid-related MYB and bHLH TFs (GmMYB173, VvMYBF1); Ca^2+^-responsive phenylpropanoid enzymes; and melatonin flavonoid modules	Soybean under NaCl; grapevine and transgenic *Arabidopsis*; CaCl_2_ or CaP-NP treatments; and melatonin-treated grape, tomato, and Brassica under salinity.	Flavonoids act both downstream and upstream of ABA, JA, IAA, ethylene, and MT signaling, feeding back on hormone biosynthesis and Ca^2+^-dependent CDPK/MAPK cascades to coordinate antioxidant defense, ion homeostasis, and growth responses.	[25,26,110,115,116]

## 5. Genetic Engineering and Breeding Applications

**Figure 4:**
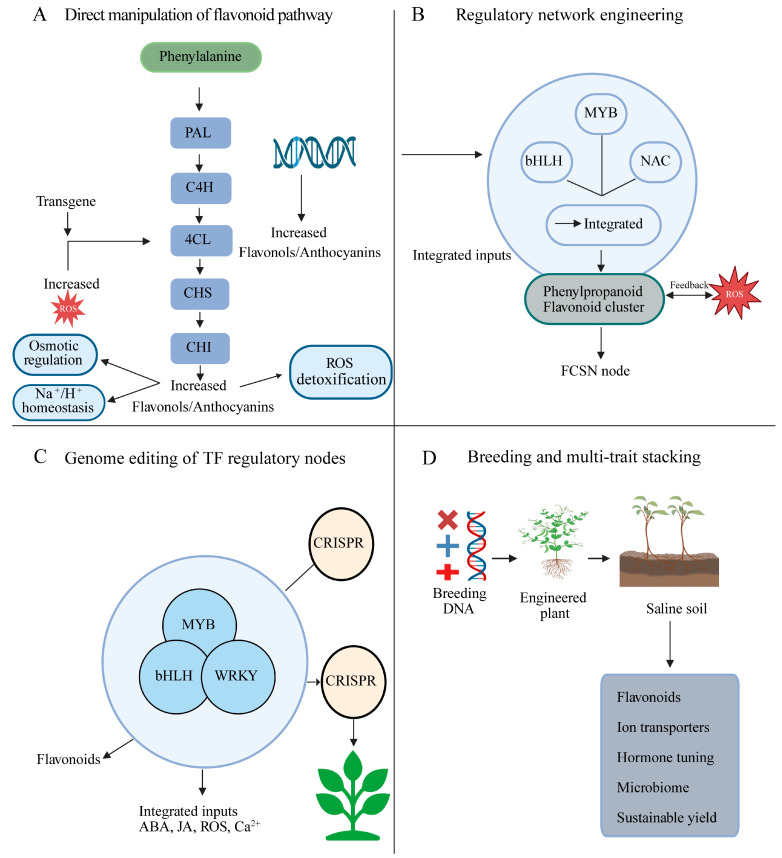
Genetic engineering and breeding strategies targeting flavonoid-centered salt stress networks. (**A**) Direct manipulation of structural genes (CHS, CHI, DFR/FLS) to boost flavonol/anthocyanin production. (**B**) Transcription factor network integration (MYB-bHLH-NAC) coordinating phenylpropanoid flux into FCSN nodes, (**C**) CRISPR-based regulatory rewiring enabling multi-layer stress-responsive control, and (**D**) multi-trait stacking via breeding and engineered plants combining flavonoids, ion transporters, hormone tuning, and rhizosphere microbiome for enhanced salinity tolerance.

A growing understanding of flavonoids as central hubs in salt stress networks now supports the rational design of salinity-resilient crops. Moving from mechanism to application, this section outlines how genetic engineering and breeding approaches can leverage flavonoid pathways and their regulators and how these strategies can be integrated into sustainable agriculture. Figure 4 summarizes practical genetic engineering and breeding routes to enhance salinity tolerance by targeting flavonoid pathway regulators and structural genes and their integration with signaling and rhizosphere modules.

### 5.1. Direct Manipulation of Flavonoid Biosynthetic Pathways

The most direct strategy is to increase flux through flavonoid branches that support ROS buffering, ion homeostasis, and membrane protection under salt stress.

#### 5.1.1. Transgenic Overexpression of Structural Genes

Constitutive or stress-inducible overexpression of core enzymes reliably raises flavonoid pools and improves stress tolerance. In rice, *F3H* overexpression increases quercetin and kaempferol, limits oxidative damage, and enhances performance under 150 mM NaCl [96]. In *Arabidopsis* and tobacco, overexpression of *CHS* or *CHI* orthologs elevates flavonoids, reduces H_2_O_2_ and MDA, and enhances tolerance to salinity and drought [90,91]. Similar effects have been observed for other steps in the pathway. *AeCHS* from *A. esculentus* and *EkFLS* from *E. kansui* increase flavonoid levels and salt tolerance when expressed in Arabidopsis [90,144]. Tailoring enzymes at branch points further refines flux. In *M. truncatula*, β-glucosidase MtBGLU17 overexpression elevates flavonoid accumulation and improves salt/drought tolerance by enhancing antioxidant activity [145]. Similarly, in rice, the UDP-glucosyltransferase GSA1 redirects phenylpropanoid carbon from lignin to flavonoid glycosides, improving tolerance to multiple stresses, including salinity, and increasing grain size [146]. UGT76E11 and CrUGT87A1 overexpression in Arabidopsis likewise boosts flavonoid glycosides and enhances NaCl tolerance [66,86].

Overall, boosting structural genes and UGT activities increases flavonoid pools and improves salt resilience through enhanced ROS scavenging and Na^+^ tolerance. However, single-gene overexpression may also uncover metabolic bottlenecks, subtly modify lignin or cell wall composition, or lead to undesired pigmentation, which, together, highlights the need for more precise pathway engineering.

#### 5.1.2. Precision Genome Editing for Pathway Engineering

CRISPR-Cas technologies enable fine-tuned rewiring of flavonoid metabolism in crops such as rice [147], tomato [148], grapevine [149], and apple [150]. Editing anthocyanin genes (*OsF3′H*, *OsDFR*, *OsLDOX*, *OsTTG1*) in rice [147] or MYB/structural loci in tomato [148] generates predictable shifts in pigment and flavonoid profiles. However, a key emerging strategy is the removal of negative regulators. CRISPR/Cas9 knockout of the MYB repressor FtMYB45 in tartary buckwheat increases rutin and total flavonoids and enhances antioxidant capacity [151]. Loss of VvbZIP36 in grapevine elevates anthocyanins [149] and in *Sesuvium portulacastrum*, salt-induced modulation of flavonoid glycosylation and methylation supports oxidative protection and ion homeostasis [104], highlighting the functional importance of post-synthetic flavonoid modification as a targetable trait. In rice, CRISPR/Cas9-targeted mutagenesis of OsRR22 improves salinity tolerance [152], and knockout of OsbHLH024 enhances salt stress resistance [153]. In soybean, CRISPR/Cas9 mutation of *GmAITR* family genes increases salinity stress tolerance [154]. In *Arabidopsis*, UGT86A1 loss-of-function mutants are salt-sensitive, whereas overexpression lines show enhanced tolerance, supporting a role for glycosylation-linked metabolite homeostasis in abiotic stress adaptation [155]. Similar edits in MYB repressors of flavonol biosynthesis raise flavonols without severe growth penalties in pepper [156]. Genome editing can reprogram flavonoid flux and modification. Notably, NaCl-phenotyped edits (OsRR22, OsbHLH024, *GmAITR*, and UGT86A1) improve salinity tolerance, which is consistent with enhanced ROS control, membrane stability, and ionic/osmotic homeostasis. In contrast, edits such as VvbZIP36, GmUGT, and pepper MYB repressors mainly demonstrate pathway controllability.

Beyond knockout-based approaches, functional studies of key regulators such as *OsTTG1* demonstrate how transcriptional control reshapes anthocyanin and flavonoid biosynthesis in rice, providing a regulatory framework that complements emerging genome editing strategies [147,157]. Multiplex editing acknowledges the polygenic nature of flavonoid flux: simultaneous targeting of *GmF3H1*, *GmF3H2*, and *GmFNS-1* in soybean boosts isoflavones and alters stress-related traits [158], while *F3′H* knockout in poinsettia (*Euphorbia pulcherrima*) redirects flux to pelargonidin [159]. Viewed in the context of salt stress, these genome editing strategies are well suited to building salt-inducible flavonoid modules, enabling quantitative tuning of pathway output rather than simple on/off control.

### 5.2. Engineering Regulatory Networks and Transcription Factors

#### 5.2.1. The “Master Switch” Strategy

Targeting master regulators (R2R3-MYB, bHLH, NAC) offers broader and more coordinated rewiring than manipulating single enzymes. Overexpression of *AtMYB12* in Arabidopsis [23] and *VvMYBF1* in grapevine [25] or *GmMYB12* in soybean [160] increases expression of PAL, CHI, F3H, FLS, and DFR, elevates flavonoids, enhances antioxidant enzyme activities, and lowers MDA/H_2_O_2_ under salt. bHLH factors such as AmDEL [161] and SibHLH22 [161] likewise drive flavonoid accumulation and salt tolerance, sometimes with growth penalties like reduced leaf size or dwarfism. These transcription factors function as key integrators, processing hormonal (ABA, JA), redox (ROS), and ionic (Ca^2+^) inputs and modulating downstream antioxidant capacity, stomatal behavior, and ion transport. Engineering such “master switches” is, therefore, a form of network-level engineering rather than just boosting a single antioxidant pathway.

#### 5.2.2. Synthetic Biology and Customized Regulation

Synthetic biology is moving towards on-demand flavonoid production optimized for agronomic performance. Synthetic promoters responsive to salinity or ABA can restrict TF or biosynthetic gene expression to stress periods and vulnerable tissues (e.g., root epidermis, young leaves), improving resource use efficiency and avoiding costs of constitutive accumulation [162,163]. Orthogonal circuits, such as engineered receptors or synthetic TFs that couple osmotic/ionic sensing directly to flavonoid output, could bypass parts of the endogenous network to generate rapid and specific protective responses [163,164]. Such designs graft an external control layer onto endogenous stress and flavonoid-signaling circuits, allowing for the precise temporal and spatial deployment of flavonoid-based protection without permanently diverting resources from growth.

### 5.3. Integration with Breeding and Multi-Trait Stacking

#### 5.3.1. Marker-Assisted and Genomic Selection

Natural allelic variation in structural and regulatory genes (*CHS*, *FLS*, *MYB*, *UGTs*) underlies heritable diversity in flavonoid profiles and salt responses [163]. These loci can serve as markers in marker-assisted selection (MAS) to pyramid favorable alleles into elite germplasm [165]. Genomic selection models that incorporate flavonoid-related loci alongside QTLs for ion homeostasis and yield may help breed cultivars with inherently stronger antioxidant capacity and salinity tolerance [156,166]. This is especially attractive in regions where GM/GE crops face regulatory or public acceptance barriers and where adaptive alleles already exist in landraces and wild relatives from saline environments.

#### 5.3.2. Rational Trait Stacking for Synergistic Resilience

Durable salinity tolerance will require stacking complementary traits. Promising combinations include flavonoid modules that enhance stress-inducible flavonols/anthocyanins or TFs that tightly couple ABA/JA signals to specific flavonoid branches [42,167]. Additional complementary modules include ion–homeostasis mechanisms (NHX, HKT, SOS1) for vacuolar Na^+^ sequestration and xylem Na^+^ retrieval [168]. Hormonal nodes such as edited ABA receptors or DREB/AREB regulons balance stress responses with growth [169]. In such pyramids, flavonoids limit oxidative and osmotic damage, while ion transport and hormonal modules directly restrain Na^+^ toxicity and support water relations, yielding more comprehensive and stable resilience [93,170].

### 5.4. Implications for Sustainable Agriculture and Crop Resilience

Flavonoid-centric engineering has clear implications for sustainable agriculture. Crops with well-integrated flavonoid-based stress responses can maintain productivity on saline or marginal lands, reducing pressure to expand agriculture into natural ecosystems and lowering the need for freshwater leaching. This strategy represents an “intrinsic biofortification” of stress tolerance, strengthening endogenous defenses and potentially reducing reliance on external chemical inputs [171]. Elevated flavonoid content may simultaneously improve nutritional and functional qualities of plant products, adding economic value [172].

The structuring role of flavonoid-rich root exudates in assembling beneficial rhizosphere microbiomes further extends benefits to soil health and nutrient cycling. However, regulatory constraints, public perception of GM/GE crops, and the complexity of network-level manipulations remain major challenges [171,173]. Engineered traits must be validated across environments and genetic backgrounds to avoid yield penalties or context-dependent failures. Overall, the transition from understanding flavonoid biology to designing flavonoid-informed crops is already underway. Viewing flavonoids as central nodes in an integrative stress network rather than as simple antioxidants enables network-aware engineering of crops with a built-in and finely tuned capacity to withstand salinity, contributing to a more resilient and sustainable global food system.

## 6. Flavonoid-Centered Integration of Redox, Hormonal, and Metabolic Networks Under Salt Stress

### 6.1. Coordination Between Redox and Hormonal Networks

Salt stress perturbs both cellular redox status and phytohormone signaling, and flavonoids operate at their intersection [174,175]. Excess NaCl drives ROS production in multiple organelles and the apoplast while inducing ABA and other growth regulators. ABA promotes stomatal closure and antioxidant defenses (SOD, CAT, APX, GR), but is itself reinforced by ROS, creating a bidirectional ABA-ROS loop [38]. Within this interaction, a flavonoid-ROS-ABA triangle can be envisaged. ABA and ROS activate the phenylpropanoid–flavonoid pathway, increasing phenolic acids, flavonols, and anthocyanins in roots and shoots [33,176]. These compounds act as non-enzymatic antioxidants that cooperate with AsA-GSH metabolism to buffer ROS, stabilize photosystems, and protect membranes. In turn, reduced ROS levels produce feedback on ABA biosynthesis, catabolism, and downstream TFs (DREB, AREB/ABF, MYC/MYB), thereby closing the loop and positioning flavonoids as both products and regulators of ABA-ROS signaling [177,178]. Beyond detoxification, this “buffering” can shape ROS signal amplitude and duration, thereby influencing whether ROS acts primarily as damage or as a second messenger in ABA-driven responses. In biochemical terms, this positions flavonoids as “redox rheostats” that tune ROS signatures (magnitude, lifetime, and compartmentation) rather than simply eliminating ROS.

This ABA module is embedded in wider JA/ET and auxin networks. JA-Ile perception by the SCF-COI1-JAZ complex releases TFs such as MYC2 under stress, which coordinately regulate stress genes and flavonoid biosynthetic genes (*PAL*, *F3′H*, *FLS*, *LAR*), thereby coupling JA signaling to flavonoid-dependent redox buffering [40,42]. Flavonols, by modulating PIN-dependent auxin transport, reshape auxin gradients that underlie root elongation, lateral root formation, and halotropism and, thus, help define spatial patterns of growth maintenance versus arrest in saline soils [47]. This provides a mechanistic “integration point” in which flavonoids translate redox/hormone status into spatial growth decisions through PIN-mediated auxin redistribution rather than only changing total hormone levels. Accordingly, flavonoids influence downstream growth programs by altering auxin distribution patterns that control cell expansion and differentiation under salt stress.

Ca^2+^ signaling provides a further integrative layer within the hormonal network. Salt-induced ROS and membrane depolarization trigger Ca^2+^ influx and systemic Ca^2+^ waves decoded by CDPKs, CCaMKs, and phosphatases that impinge on ABA, JA, and phenylpropanoid targets [179]. Apoplastic H_2_O_2_ is sensed by receptors such as GHR1 and HPCA1, linking ROS-Ca^2+^ dynamics to stomatal control and systemic acclimation [180,181]. In this context, flavonoids function as local ROS buffers, constraining H_2_O_2_ amplitudes without abolishing signaling, thereby indirectly shaping Ca^2+^ signatures and hormone responses [75,182]. Thus, flavonoids can support “signal integration” by maintaining signal-grade ROS/Ca^2+^ dynamics while preventing runaway oxidative injury. Because Ca^2+^ signatures are decoded into transcriptional outputs through kinase/phosphatase modules, flavonoid-dependent redox buffering can influence the interpretation of Ca^2+^ waves and thereby bias downstream gene expression programs under salinity. These findings support a model in which flavonoids coordinate ROS buffering with ABA, JA/ET, SA, auxin, and Ca^2+^ signaling during salt stress.

### 6.2. Metabolic Reprogramming and Cellular Energy Balance

Salinity forces a reallocation of carbon and energy from growth to defense. Reduced photosynthetic performance and mitochondrial efficiency shift metabolism towards glycolysis and soluble sugar accumulation for ATP supply and osmoprotection [183,184]. Multi-omics studies consistently show that a portion of this carbon is channeled into phenylpropanoid and flavonoid biosynthesis, despite the metabolic cost, because these compounds provide high-capacity ROS scavenging, stabilize the AsA-GSH cycle, and modulate hormone signaling [181,185]. From a metabolic perspective, flavonoids represent a high-return investment under salt stress: even modest carbon allocation to flavonoid biosynthesis can stabilize redox balance, protect membranes, and maintain transporter function, thereby supporting continued carbon assimilation.

Physiological examples illustrate this investment logic. In Paulownia, NaCl increases flavonoids and proline, with enhanced antioxidant capacity and only moderate declines in PSII efficiency, indicating joint protection of thylakoid membranes and electron transport [134]. In *G. biloba*, methyl jasmonate co-treatment under salinity enhances flavonoid-related gene expression, increases quercetin content, and limits oxidative damage, demonstrating hormone-driven re-channeling of carbon into flavonoid pools that stabilize chloroplast function [143]. Across species, salt and/or ABA commonly upregulate *PAL*, *CHS*, *F3H*, *FLS*, *ANS*, and *UFGT*, increase phenolic acids and flavonoids, and raise total antioxidant capacity, thereby supporting both redox homeostasis and ion/osmotic balance [33,186].

Post-synthetic modification further optimizes this investment. Glycosylation by stress-inducible UGTs, such as UGT76E11 in *Arabidopsis*, maintains flavonoid pools under salt and drought, generating long-lived glycosides that can be safely stored or transported while retaining antioxidant and signaling functions [86]. At the root–soil interface, increased secretion of flavonoid-rich exudates under salinity promotes the recruitment of plant-growth-promoting bacteria and other beneficial taxa that support ion balance, hormone adjustment, and antioxidant capacity [137,187]. Because modification and transport determine where flavonoids accumulate (vacuole, apoplast, vascular stream, rhizosphere), these steps are not “downstream decoration”, but part of how flavonoids execute spatial control over stress physiology. Thus, flavonoid flux represents a carbon investment into both internal buffering and external microbial partnerships, contributing to sustained growth and reproduction under chronic salinity. Noncoding RNAs add a post-transcriptional regulatory layer to flavonoid-mediated salt responses. miRNAs that target R2R3-MYB regulators can rapidly reprogram phenylpropanoid–flavonoid gene expression and flavonoid accumulation [188]. lncRNAs further tune stress transcription in cis/trans; in Arabidopsis, DRIR enhances drought/salt tolerance and modulates ABA-responsive gene programs [189]. In *G. biloba*, the salt-responsive LncNAT11-MYB11-F3′H/FLS module promotes flavonol biosynthesis and improves NaCl tolerance, linking an lncRNA regulator to a defined flavonoid branch and a measurable phenotype [24]. These findings support incorporating ncRNA control into flavonoid-centered models of signal integration [190].

### 6.3. Flavonoid-Centered Stress Network (FCSN): A Conceptual Model

**Figure 5:**
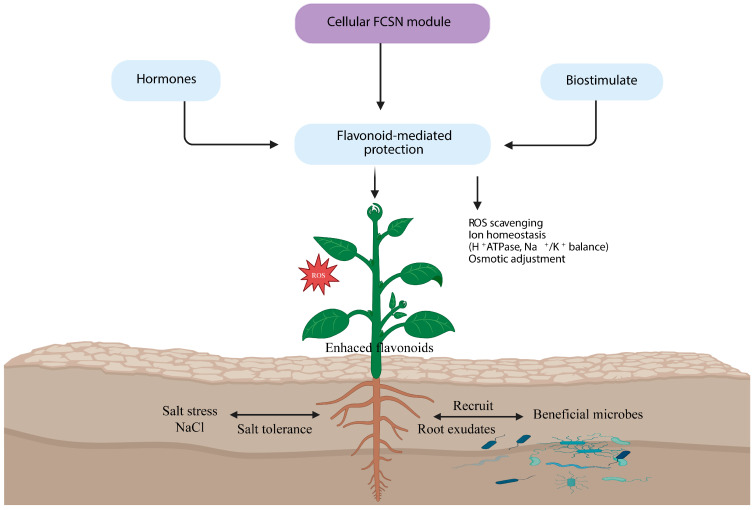
Standalone block diagram of the flavonoid-centered stress network (FCSN) integrating metabolism, hormones, signaling, and post-synthetic modification layers across scales (cell-tissue, whole-plant, and rhizosphere scales). The diagram refines and integrates the cellular network logic summarized in Figure 2 with whole-plant outcomes and rhizosphere feedback via flavonoid-rich root exudates. Core modules include (i) metabolic flux and pathway branching in the phenylpropanoid–flavonoid pathway, (ii) hormone crosstalk (ABA, JA, ethylene, and auxin), (iii) Ca^2+^/ROS and kinase signaling (MAPKs/CDPKs) with redox feedback, and (iv) post-synthetic modification and spatial control (glycosylation/acylation, transport, and compartmentation), culminating in measurable tolerance phenotypes under salinity.

From a systems perspective, flavonoids are not merely terminal products of stress metabolism, but function as key nodes that interlink and stabilize redox, metabolic, and signaling networks. On this basis, we propose a conceptual model, the flavonoid-centered stress network (FCSN). To apply this framework, we present a standalone block diagram (Figure 5) that integrates and refines the cellular-scale network shown in Figure 2 and maps it across cell-tissue, whole-plant, and rhizosphere scales.

At the cellular scale, the FCSN is organized into four explicitly connected layers: (i) metabolism (phenylpropanoid–flavonoid flux and pathway branching), (ii) hormones (ABA, JA, ethylene, and auxin and associated transcriptional modules), (iii) signaling (Ca^2+^/ROS signatures and kinase cascades such as MAPKs and CDPKs), and (iv) post-synthetic modification and spatial control (glycosylation/acylation, transport, and compartmentation). Bidirectional interactions among these layers allow flavonoid pools to function as integrative nodes that couple upstream salt perception to downstream physiological outputs.

At the tissue and organ scale, the FCSN indicates patterned flavonoid accumulation and signaling. In leaves, flavonoids in epidermis and mesophyll protect PSII, preserve pigments, and refine stomatal behavior; in roots, local flavonoid induction shapes auxin gradients, endodermal barriers, and Na^+^ handling, contributing to adaptive root architecture and ion homeostasis.

At the whole-plant and rhizosphere scales, integration of these modules yields emergent properties: stable photosynthetic carbon supply, efficient Na^+^ exclusion or compartmentation, flexible root system architecture, and a flavonoid-structured microbiome that enhances nutrient acquisition and stress resilience. External inputs such as ABA, MeJA, and PGPB can be viewed as network modulators that selectively reinforce parts of the FCSN-hormonal hubs, phenylpropanoid flux, and exudation patterns without altering its core architecture.

This conceptual model may have practical implications. Rather than treating flavonoids as generic antioxidants, breeding, metabolic engineering, and bio-stimulant strategies can deliberately target specific flavonoid branches and their regulatory nodes within the FCSN. Such network-aware manipulation is likely to produce more robust and predictable improvements in salinity tolerance than approaches that ignore the significant role of flavonoids in coordinating redox, metabolic, and signaling networks. This integration from the cellular to the rhizosphere level is depicted in Figure 5.

On this basis, testable predictions derived from the FCSN framework include the following: (1) Increasing flux into protective flavonoid branches should reduce ROS injury (H_2_O_2_/MDA) and electrolyte leakage and improve Na^+^/K^+^ homeostasis under matched salt regimes. (2) Perturbing post-synthetic modification (e.g., glycosylation/acylation) should shift the spatial pattern of protection and weaken whole-plant tolerance even when total flavonoids change modestly. (3) Disrupting signaling hubs (Ca^2+^/ROS–MAPK/CDPK nodes) should decouple flavonoid induction from downstream ion transport outputs. (4) Enhancing flavonoid export/exudation should measurably alter rhizosphere community composition and, if a feedback loop operates, the tolerance benefit should be reduced in microbiome-depleted conditions.

## 7. Conclusions and Future Perspectives

### 7.1. Conclusions

Salt stress simultaneously perturbs water relations, ion homeostasis, redox balance, and developmental programs. The studies synthesized in this review show that flavonoids contribute to salt tolerance at multiple levels, extending far beyond a simple role as non-enzymatic antioxidants. Under salinity, transcriptional, hormonal, and epigenetic regulation remodels the phenylpropanoid–flavonoid pathway, leading to the accumulation of specific flavonoids and their derivatives that buffer ROS, support Na^+^/K^+^ balance, stabilize membranes, and modulate hormone and Ca^2+^ signaling. Moreover, spatially patterned accumulation in roots, leaves, and the rhizosphere, together with flavonoid-mediated adjustment of auxin fluxes and root exudation, links local stress perception to systemic acclimation. On this basis, we conceptualized a flavonoid-centered stress network (FCSN) in which flavonoids act as integrative nodes connecting biosynthetic, redox, and signaling modules across cellular, tissue, and whole-plant scales. Viewing flavonoids through this network lens helps explain their recurrent association with salt tolerance in both glycophytes and halophytes and provides a theoretical framework for targeted crop breeding to improve salinity resilience.

### 7.2. Challenges and Constraints in Exploiting Flavonoids for Salt Tolerance

Despite clear advances, several issues still limit the translation of flavonoid knowledge into breeding and engineering:

(i) Metabolic trade-offs:
  Flavonoid synthesis is carbon/energy costly; constitutive accumulation can reduce growth/yield.
  Stress-inducible, tissue-specific, or developmental control are preferable to minimize penalties.
(ii) Specificity and context dependence:
  Effects depend on flavonoid class, localization, and dose; different branches can have opposing outcomes.
  Responses vary across species/genotypes and under combined stresses (salt + drought/heat).
(iii) Pleiotropy from pathway crosstalk:
  Flavonoids intersect with lignin, auxin transport, hormone balance, and cell wall traits.
  Editing enzymes/TFs may alter architecture or mechanics; agronomic evaluation is essential.
(iv) Regulatory uncertainty:
  Epigenetic regulation and ncRNAs influence both flavonoids and salt response pathways.
  These controls are dynamic and can vary across environments and generations.
(v) Limited field validation:
  Many studies use seedlings and controlled NaCl regimes.
  Stability under fluctuating salinity, heterogeneous soils, and multi-stress field settings remain under-tested.

### 7.3. Future Research Directions

Building on the FCSN concept, several key directions emerge for future work.

More work is needed to distinguish the roles of individual flavonoids (e.g., quercetin vs. kaempferol glycosides, C-glycosylflavones, proanthocyanidins) and to link these functions to specific cell types and compartments. Combining single-cell omics, advanced imaging, and targeted metabolite profiling will help map where and when flavonoids are required during salt exposure.

How DNA methylation, histone modifications, siRNAs, and lncRNAs coordinate flavonoid biosynthesis with ion transport, hormone signaling, and ROS homeostasis under recurrent or chronic salinity is still poorly understood. Integrating these layers into FCSN-oriented models could improve prediction of stress “memory” and transgenerational responses. Genome editing, synthetic promoters, and engineered transcriptional circuits can be used to couple salt, ABA, or JA signals to specific branches of the flavonoid pathway. Priority targets include stress-inducible activation of high-capacity antioxidant flavonols/anthocyanins or the context-specific modulation of flavonoid-mediated auxin transport while minimizing off-target developmental effects.

Flavonoid-based strategies should be integrated with improvements in Na^+^ exclusion/compartmentation (e.g., SOS, HKT, NHX) and with optimized ABA/JA signaling modules. Testing such multi-trait stacks in diverse genetic backgrounds will help determine how FCSN components interact with classical salt tolerance mechanisms. Flavonoid-rich root exudates shape root-associated microbial communities. Future work should examine how engineered changes in exudate composition affect the recruitment of plant-growth-promoting microbes, ion-cycling consortia, and disease-suppressive communities in saline soils and how these microbiome shifts feed back into the FCSN.

Finally, the FCSN model should be tested and refined using multi-omics and physiological datasets from field trials across salinity gradients. Such studies will clarify which network motifs are most predictive of stable tolerance and identify practical biomarkers (metabolites, gene expression signatures) for selection in breeding programs.

## Data Availability

Not applicable.
